# Parameter-sweeping techniques for temporal dynamics of neuronal systems: case study of Hindmarsh-Rose model

**DOI:** 10.1186/2190-8567-1-6

**Published:** 2011-07-11

**Authors:** Roberto Barrio, Andrey Shilnikov

**Affiliations:** 1 Departamento de Matemática Aplicada and IUMA, Universidad de Zaragoza, E-50009, Zaragoza, Spain; 2 Neuroscience Institute and Department of Mathematics and Statistics, Georgia State University, Atlanta, Georgia, 30303, USA

## Abstract

**Background:**

Development of effective and plausible numerical tools is an imperative task for thorough studies of nonlinear dynamics in life science applications.

**Results:**

We have developed a complementary suite of computational tools for two-parameter screening of dynamics in neuronal models. We test a ‘brute-force’ effectiveness of neuroscience plausible techniques specifically tailored for the examination of temporal characteristics, such duty cycle of bursting, interspike interval, spike number deviation in the phenomenological Hindmarsh-Rose model of a bursting neuron and compare the results obtained by calculus-based tools for evaluations of an entire spectrum of Lyapunov exponents broadly employed in studies of nonlinear systems.

**Conclusions:**

We have found that the results obtained either way agree exceptionally well, and can identify and differentiate between various fine structures of complex dynamics and underlying global bifurcations in this exemplary model. Our future planes are to enhance the applicability of this computational suite for understanding of polyrhythmic bursting patterns and their functional transformations in small networks.

## 1 Introduction

 Individual and networked neurons can generate various complex oscillations known as bursting, formed by alternating fast repetitive spiking and quiescent or subthreshold oscillatory phases. Bursting is a manifestation of composite, multiple time scale dynamics observed in various fields of science as diverse as food chain ecosystems, nonlinear optics, medical studies of the human immune system, and neuroscience. The role of bursting is especially important for rhythmic movements determined by Central Pattern Generators (CPG). Many CPGs can be multifunctional and produce polyrhythmic bursting patterns on distinct time scales, like fast swimming and slow crawling in leeches [1]. Such CPGs are able to switch between different rhythms when perturbed [2,3].

In mathematical neuroscience a deterministic description of endogenously oscillatory activities, like two-time scale bursting, is done by revealing generic properties of mathematical and realistic models of neurons; the latter are derived through the Hodgkin-Huxley formalism for gating variables. Either bursting model falls into a class of dynamical systems with at least two time scales, known as slow-fast systems.

 Configurations and classification schemes for bursting activities in neuronal models first proposed in [4] and extended in [5,6] are based on geometrically transparent mechanisms that initiate and terminate the so-called slow motion manifolds composed of the limiting solutions, such as equilibria and limit cycles, of the fast subsystem of a model [7-11]. These manifolds constitute the backbones of bursting patterns in a neuronal model. A typical Hodgkin-Huxley model possesses a pair of such manifolds [4]: quiescent and tonic spiking. The existing classifications of bursting are based on codimension-one bifurcations that initiate or terminate the fast trajectory transitions between such one-dimensional [1D] and two-dimensional [2D] slow motion manifolds in the phase space of a model. These classifications single out the classes of bursting by subdividing neuronal models into the following types: elliptic or Hopf-fold; square-wave burster, or fold-homoclinic; parabolic, or circle-circle class describing top-hat models. These terms are either due to specific shapes of voltages traces in time, or after the static underlying bifurcations that occur in the fast subsystem of the given neuron model.

**Fig. 1:**
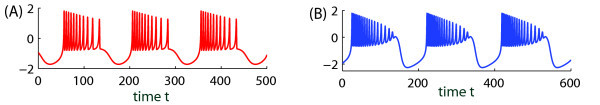
**(A)** Square-wave and **(B)** plateau-like bursting traces in the Hindmarsh-Rose model at b=2.7 and 2.52, respectively. Transformations of bursting can be detected quantitatively by a sudden change of the number of spikes per burst.

**Fig. 2:**
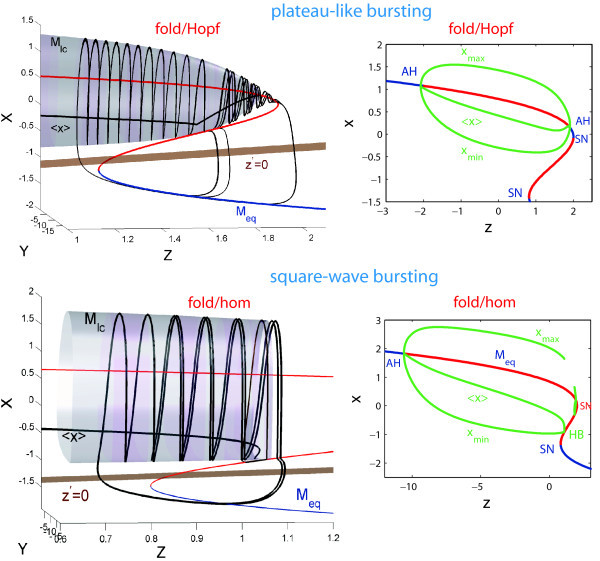
Top: a plateau-like or fold/Hopf bursting starts after the spiking manifold $M_{\mathrm{lc}}$ becomes tangent to the middle, saddle branch of $M_{\mathrm{eq}}$ and terminates further through the reverse supercritical Andronov-Hopf bifurcation on the upper depolarized branch of $M_{\mathrm{eq}}$. Bottom: the primary feature of the square-wave bursting activity in the HR model also referred to as of fold/homoclinic type is the termination of the spiking manifold $M_{\mathrm{lc}}$ by the homoclinic bifurcation in the phase space of the fast subsystem. In both cases: fold stands for a saddle-node bifurcation at the turning point (SN) on the lower, hyperpolarized branch of $M_{\mathrm{eq}}$.

The types of the static bursting configurations in the Hindmarsh-Rose model shown in Figures 1 and 2 are also named *fold/homoclinic* and *fold/Hopf*, as this would indicate that the terminal phases of the fast spiking and slow quiescent periods are determined, respectively, by a homoclinic bifurcation of a saddle equilibrium state, or a supercritical Andronov-Hopf bifurcation, along with a saddle-node bifurcation of equilibria, respectively, which all occur in the fast subsystem of the model. In the next section we will examine the transition bifurcation patterns between these types of bursting. 

These manifolds, especially their stable branches, can be easily traced and visualized in the phase space by utilizing the slow variable as a sweeping parameter in the decoupled fast subsystem. Far from bifurcations, this slow-fast dissection approach allows for exhaustive simplifications that let one treat the dynamics of the full model as on overlay the uncorrelated dynamics of its fast subsystem mediated by repetitive passages of the slow variable.

 The slow-fast dissection has been proven to work very well for a low-order model of a bursting neuron *as long as* it stays away from a bifurcation that is due to reciprocal interactions of the dynamics of both subsystems. Such a bifurcation, underlying a bursting transition(s), gives rise to the emergence of dynamical phenomena that can *only* occur in the full system. For example, this occurs when the dynamics of the fast subsystem falls to the time scale of the slow subsystem, particularly near saddle-node and homoclinic bifurcations. A classic example is the onset of chaotic dynamics of finite subshift shown by D. Terman [12] at the transition between tonic spiking and bursting that happens to be generic for a square-wave burster like the Hindmarsh-Rose model [13-15]. In addition [12] gives an explanation of common spike adding cascade in classical square-wave bursters which is due to a slow passage of the phase point near the saddle in the fast subsystem. Note that the nature of spike adding cascade could be bifurcationally different, as for example in the leech interneuron model due to the blue sky catastrophe [16] prolonging the burst duration phase, or because of homoclinics of a saddle periodic orbit [17,18] playing the role of the chaotic potential barrier, loosely speaking, that bursting should overcome to get an extra spike.

 Complex dynamics, including quick period doubling cascades in square wave bursters [19,20] can also be explained in terms of codimension-two homoclinic bifurcations, including inclination-switch and orbit-flips, that occur at the transition [21,22] from tonic spiking to bursting. Recent breakthrough examples of novel transitions to and from bursting due to reciprocal interactions of slow-fast dynamics include various homoclinic of saddles and saddle-foci, the blue sky catastrophe, bistability due non-transverse homoclinics to saddle-node periodic orbits, canard-tori [13,16-18,23-26]. The range of bifurcations and dynamical phenomena giving rise to bursting *transcends* the existing static classification schemes based solely on slow-fast dissection.

In-depth understanding of the generic mechanisms combined into a broad global picture on the transition patterns between the activity types in typical models of individual neurons presents a fundamental challenge for the theory of applied dynamical systems. In response to variations of intrinsic parameters, or an external applied current, like *I* in (1), a neuron model should demonstrate, migrate, and switch flexibly between various types of activities such as quiescence, tonic spiking and bursting. In addition, nonlinearity of the model can often imply bi- or multi-stability of several co-existing activities at the same parameter values. Bistability of coexisting oscillatory patterns originate near global bifurcations taking place in the model. Multistability is well noticeable when a targeted activity can be robustly selected by choosing other initial conditions or by temporal perturbations, like applied external current. Having ascertained such a global picture we can make consistent predictions for determining basic principles of the functioning of coupled neurons on networks where they receive mixed, inhibitory and excitatory perturbations from other neurons and synergetically reciprocate.

The applied dynamical systems community has developed a universal and versatile suite of computational tools and techniques for comprehensive examination of diverse nonlinear systems of various origins including life sciences. These tools allow a researcher to mono- and bi-parametrically scan the model in question to search for specific transformations corresponding to local and global bifurcations that often hard to detect by standard means. One approach to an initial examination of an unknown model, often called ‘brute-force’ approach, is to evaluate the largest Lyapunov exponent. This approach has long been widely utilized in nonlinear dynamics for initial detection of bifurcations of steady state and oscillatory solutions. The brute-force approach contrasts drastically from examination of fine bifurcation structure of *limit* solutions of the system. Nevertheless, if performed extensively the brute force approach reveals adequately the backbone of the bifurcation structure of the model, which can be further enhanced and complemented with detailed bifurcation analysis that would provide the finishing touches, in the form of bifurcations curves, to the initial brute force diagram. We point out that transformations reshaping bursting waveforms en route toward the tonic spiking activity are caused by atypical bifurcations due to the presence of two, or more time scales in bursting. Because of that bifurcations of stiff bursting solutions, especially irregular ones, are hard to trace down by parameter continuation software packages such as CONTENT and AUTO-based packages, which are specifically designed mainly for explorations of equilibria and ‘typical’ periodic orbits.

 The primary goal of this paper is to demonstrate that straightforward methods used in neuroscience experimental studies can be as effective as conventional tools, based on the bifurcation and Lyapunov exponent theory, employed in nonlinear dynamics studies. In this paper, we revisit and examine transformations of various oscillatory activity types in the phenomenological Hindmarsh-Rose model of bursting neurons, viewed, so to speak, through the prism of neuroscience plausible methods. Next we compare our findings with the results obtained using the evaluation of a maximal Lyapunov exponent that was presented in detail in [27,28] which we consider as an etalon. More specifically, as a part of the comparison test, we place next to each other the bifurcation diagrams found using calculus-based computational tools yielding the whole spectrum of the Lyapunov exponents for complete solutions of the model and those obtained through examinations of 1D voltage traces, which are typically available in experimental studies. We then extract various qualitative temporal characteristic of neuronal activity from non-transient fragments of such traces, including the number of spikes per regular burst, deviations of the spike numbers in case of chaotic bursting, interspike intervals, burst duration and period, and the duty cycle which is the ‘spiking’ fraction of the bursting period. By varying two control parameters of the model, we basically perform bi-parametric sweeps of its dynamics that are aimed to detect in a very straightforward manner various global bifurcations including • transitions between quiescence, tonic spiking and bursting activities including ones through various homoclinic bifurcations; • identify regular and chaotic transformation of bursting, including a change of the bursting topology accompanying square wave to plateau-like transitioning, as well as forward and backward sequences of spike-addition and -deletion, and so forth.

## 2 Materials and methods

 The phenomenological system of ODEs proposed by Hindmarsh and Rose [29,30] for modeling bursting and spiking oscillatory activities in isolated neurons is given by:

(1) $$\begin{array}{rcl} \dot{x} & = & y-ax^{3}+bx^{2}-z+I,\\ \dot{y} & = & c-dx^{2}-y,\\ \dot{z} & = & \varepsilon \left(s(x-x_{0})-z\right); \end{array}$$

here, *x* is treated as the membrane potential, while *y* and *z* describe some fast and slow gating variables for ionic currents, respectively. Slow ‘activation’ of *z* is due to the small parameter 0<ε≪1. The parameters in (1) are typically set as follows a=1c=1d=5s=4$x_{0}=-1.6$ and ε=0.01, so that regular bursting oscillations in the model at an ‘applied current’ I=4, which belongs to the square-wave type at b=2.7, and transforms to a plateau-like bursting at b=2.52, see Figure 1. Along with ‘intrinsic,’ *b*, and ‘external,’ *I*, bifurcation parameters the dynamics of the model are sensitive to variations to other parameters: *ε* being treated as a rate of activation for some current, and $x_{0}$ being viewed as a control parameter delaying and advancing the activation of the slow current in the modeled neuron. In response to variations of intrinsic parameters, or an external applied current, like *I* in (1), a neuron model should demonstrate, migrate, and switch flexibly between various types of activities such as quiescence, tonic spiking and bursting.

In this section we will brief the core of the numerical techniques employed in the analysis of the HR model. We will start with the specifics of the numerical integration of the differential equations of the model (1).

 There are a plethora of high quality numerical integrators that have been created by numerical ODEs specialists. This study utilizes a recently developed freeware library TIDES (Taylor Integrator for Differential EquationS) available at http://gme.unizar.es/software/tides[31]. TIDES is a highly adaptive software package for numerical simulations of ODE systems. While the Taylor method is one of the oldest numerical methods for solving ordinary differential equations, it is scarcely used nowadays but its use is growing in the computational dynamics community. The formulation of the method is quite simple. First consider the initial value problem $\dot{y}=f(t,y)$. The value $y(t_{i})$ of the solution at $t_{i}$ can be evaluated as $y_{i}$ of the *n*th degree Taylor series of y(t) at $t=t_{i}$ (**f** is to be a smooth or analytical function). So, denoting $h_{i}=t_{i}-t_{i-1}$

$$\begin{array}{rcl} y(t_{0}) & =: & y_{0},\\ y(t_{i}) & ≃ & y_{i-1}+f(t_{i-1},y_{i-1})h_{i}+\frac{1}{2!}\frac{df(t_{i-1},y_{i-1})}{dt}h_{i}^{2}+\cdots +\frac{1}{n!}\frac{d^{n}f(t_{i-1},y_{i-1})}{dt^{n}}h_{i}^{n}\\ & =: & y_{i}. \end{array}$$

Therefore, the problem is reduced to the determination of the Taylor coefficients $1/(j+1)!\;d^{j}f/dt^{j}$. This can be done efficiently by using automatic differentiation techniques (see details in [32]).

 The Taylor method has several unique features [32,33]. One of its features is that it explicitly provides a dense output in the form of a power series, which becomes highly useful for detecting various instantaneous events, for example moments at which a solution hits a Poincaré cross-section, reaches a voltage maximum, for example, if one need to counts spikes in bursts, and so on. In addition, the method can be formulated using the interval arithmetics, which is often employed in the computer-assisted proofs of chaos nowadays. The Taylor method also provides high-precision solutions of ODEs so demanded in studies of systems exhibiting multiple time-scale dynamics in moderately stiff systems. In this work we use a Taylor series method of order 15 with an error tolerance set to $\mathrm{TOL}=10^{-12}$ for the most of the simulations. At this tolerance level the TIDES software is as fast and slightly more accurate [31] than the code DOPRI853 developed by Hairer and Wanner [34]. Note that TIDES is a general purpose software, and so it can be apply to general ODE systems, and not only to the HR model. We note also that we have employed the Taylor series method for solving variational equations and computations of the Lyapunov exponent spectrum, which are viewed as nonstandard options for the method [35]. As remarked, in the numerical simulations it is possible to use several good general ODE solvers, but the main advantage of the Taylor method in this kind of studies is that it provides most of the requirements we need for the problem, accuracy when needed, easy events detection, direct dense output as a power series and easy implementation of variational equations.

 As mentioned above, the continuous output generated by the integrator based on the Taylor series method is able to detect accurately and effectively various instantaneous events such as whether the phase point hits a cross-section or reaches some critical value like voltage maximum/minimum, or the number of spikes per burst approaches the sought value, which is the underlying idea for the spike-counting (SC) technique [27]. In combination, the methods allows us to classify the solutions of the HR model in neuroscience terms: no spike - quiescence (convergence to a stable equilibrium point); single tonic spike - a round periodic orbit (like one around the manifold $M_{\mathrm{lc}}$); multiple spikes within a train - bursting orbit composed of alternating tonic spike and pseudo-quiescent periods, as well as distinguishing chaotic behaviors characterized by wide variations of spike numbers in burst trains. Besides, the SC-technique allows for indirect evaluations of the duty-cycle [DC] of the orbit, which is a fraction of the burst period (that is, the ratio [Burstduration]/[Burstperiod]) of regular, periodic dynamics. Long bursting implies a duty-cycle close to one, meaning that the neuron is active most of the time; on the other hand if DC is close to zero the neuron generates rare spikes, mostly staying in the quasi-quiescent state. Finally, the continuous output of the Taylor series is also used in constructing bifurcation diagrams based on the variability of intervals between the spikes.

 The other technique that we use in this paper is the computation of the Lyapunov exponents for solutions of the HR model. In bounded systems the existence of a positive Lyapunov exponent is associated with chaotic motions, and any orbit on a compact set that does not converge to an equilibrium point has at least one zero Lyapunov exponent. Accordingly, computation of the largest Lyapunov exponents yields important information about the kind of orbits present in the system. The corresponding algorithm for the computation of the Lyapunov spectrum is a combination of the classical methods proposed by [36,37] and the ability of TIDES to compute solutions of the variational equations directly. The first variational equations of the system are integrated with the identity matrix as an initial condition, creating a canonical orthonormal basis which is mapped onto a new set of vectors. In a chaotic system each vector tends to expand along the local direction of most rapid growth, so to avoid this problem the Gram-Schmidt orthonormalization process is applied. Later the Lyapunov exponents are calculated from the growth of areas given by the different propagated vectors (for more details see [37,38]).

## 3 Screening the HR model in the (b,I)-plane

**Fig. 3:**
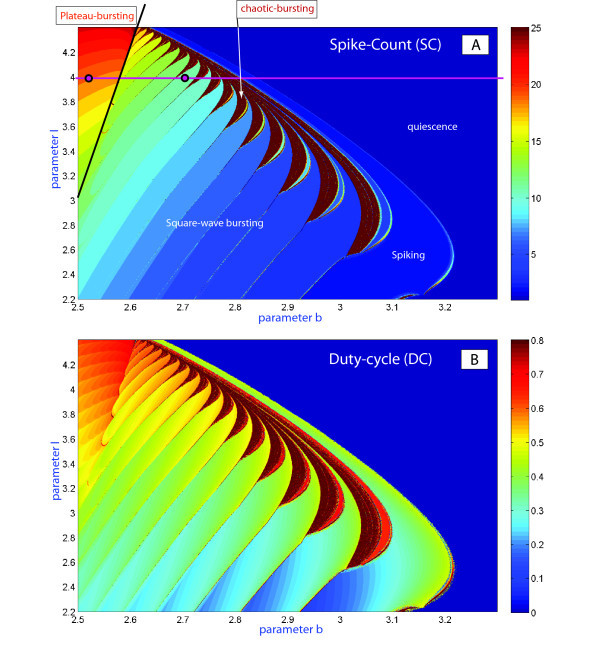
**(A)**(b,I)-parameter sweep of the Hindmarsh-Rose model based on the spike-counting approach. The color-coded bar to the right gives the spike-number range. The diagram clearly shows the boundaries of the spike-addition sequence, and the border between square-wave and plateau-like bursting. It also reveals the clove-shape structure of the zones of chaotic bursting which adjoin to the regions of tonic-spiking. **(B)** Same-range screening based on the evaluation of the the duty-cycle of bursting. The duty cycle value comes close to one near the boundary between bursting and tonic-spiking and drops close to zero near the border of the spiking region. Compare (A) and (B) with the screening diagram based on the Lyapunov exponents for solutions of the model in Figure 4 below.

**Fig. 4:**
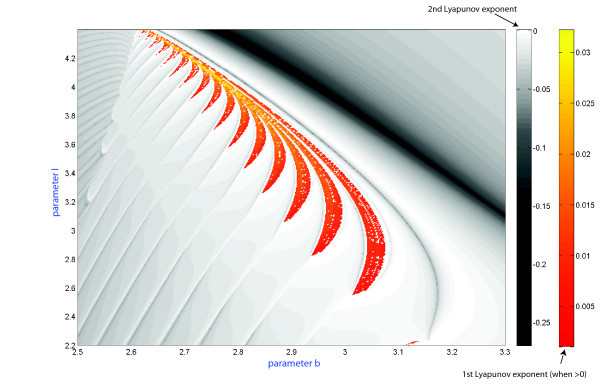
Parametric sweeping of a Lyapunov exponent spectrum: orange-colored zones indicate chaotic dynamics in the model, while a regular dynamics region is painted in grey colors of varying tint corresponding to a second Lyapunov exponent of zero value, and darker shades for negative values.

The HR model can exhibit a plethora of dynamical activities at different parameter values. Consequently, obtaining a comprehensive understanding of the multi-parametric evolution of a system like the HR model is no easy task, and hence most of the parameters are fixed in the model. In this section, the free bifurcation parameters to be varied are *b* and *I*; both are in charge of transformations for the intrinsic structure of the fast subsystem in the HR model. Next we perform the two-parameter sweep or screen of the model to collect vital data representing the time and parameter transformations of the single ‘voltage’ *x*-variable of the neuronal model. Further data mining will be carried out to extract quantitative and qualitative information about the dynamical variability of the model, bifurcations of its solutions, and so on. In Figures 3 and 4 we use a homogeneous grid comprised of 1,000×1,000 points within the given parameter range. In short, that means that this scan represents 10^6^ simulations. 

 Figure 3(A, B) presents the (b,I)-parameter spike-counting (SC) and duty-cycle (DC) diagrams of the HR model. Similar SC diagrams for the model were previously reported in [27]. The color-scale bar on the right in Inset (A) yields the number of spikes within a burst. The diagram for the duty-cycle evolution is shown in Inset (B). By combining these diagrams, we can partition the parameter plane into regions of different kinds of behaviors and classify the regimes: tonic-spiking (single spike), square-wave and plateau-like bursting, quiescence, and chaotic behaviors with the variability of spikes exceeding some preset limit. It is easy to see that both diagrams give consistent results. They reveal with clarity the region of tonic-spiking where both DC and SN take minimum values, below which there is the bursting region at the right bottom corner of the diagram. Bursting emerges from tonic spiking through the spike-addition cascade in two different ways: one is regular and reversible; the corresponding transitions are foliated by the bifurcation curves. The second kind of transitions is due to the clove-shaped regions (shown in red in Figure 4) corresponding to well-developed chaotic dynamics in the model.

 Another interesting behavioral phenomenon reshaping the type of bursting occurs in the top-left corner of the diagrams. In this region of bursting with a high number of spikes transforms into bursting with a drastically lower number of spikes per burst. To elucidate what happens on the border between these regions, we visually examined the orbits of the model. We found the border corresponding to the transition between square-wave and plateau-like bursting (see the waveforms in Figure 1 corresponding to the selected points in Figure 3(A)). The corresponding planar bifurcations underlying the robust bursting configurations of both types are well described in the literature, see [6,21]. The bursting type depends on the way the slow-motion tonic spiking manifold terminates in the phase space of the fast subsystem of the HR model. In the square-wave bursting case, the burst termination is due to a homoclinic bifurcation, also known as - fold/homoclinic, whereas in the case of plateau-like, or fold/Hopf, bursting is due to the reverse supercritical Andronov-Hopf bifurcation (Figures 1,2). In essence, this means that the parameters *b* and *I* change the structure of the fast subsystem of the HR model so that the homoclinic bifurcation is no longer transverse in the *z*-parametric cutaway in the singular limit [21]. Figure 2 elaborates on the metamorphoses of the structure transformations. One can see that plateau-like bursting takes the place of square-wave bursting after the spiking manifold, $M_{\mathrm{lc}}$, becomes tangent to the saddle branch of in the middle of $M_{\mathrm{eq}}$ and further terminates on the upper depolarized branch of $M_{\mathrm{eq}}$ through the supercritical Andronov-Hopf bifurcation. In general, this is not a cod-1 bifurcation, but a degeneracy due to loss of transversality.

 The HR model can exhibit complex, chaotic bursting with a large number of spikes, especially near transitions to hyperpolarized quiescence (see Figure 3). In context of the dynamics of the model, bursting is technically treated as chaotic if trains have more than 25 unlike spikes with distinct interspike intervals. A large number of spikes can also be generated by periodic bursting. In order to differentiate between regular and chaotic bursting behaviors, we have also used another computational technique. The complete spectrum of the Lyapunov exponents was evaluated for the orbits of the model as the two parameters were varied within the same range. In the simulations we discard a transient time of 10^3^ and we integrate till 10^5^ with the algorithm to compute the exponents and using as initial conditions the last value of the previous simulation. The corresponding sweeping diagram is shown in Figure 4. In the diagram, shown in the yellow-orange scale refers to the regions where the first Lyapunov exponent is positive. This means the occurrence of chaotic dynamics in the model. The gray-colored regions are where the second Lyapunov exponent is negative while the first Lyapunov exponent remain zero on periodic orbits. The Lyapunov exponent based diagram also reveals the spike adding transitions, and the corresponding bifurcation lines can be drawn where the 2nd Lyapunov exponent reaches the maximal value of zero. This implies that the bifurcating bursting orbit is about to disappear and will be replaced by the successive bursting orbit with an extra spike in each train. Spike adding transitions were observed and studied in several neuron models, including the Chay and Hindmarsh-Rose mathematical models [15,39,40], and the leech heart interneuron [17]. Note that there are several universal scenarios for such cascades, including saddle-node bifurcations, homoclinic bifurcations of saddle equilibria [19,41] and periodic orbits [18], as well as through the blue sky catastrophe [21,22].

**Fig. 5:**
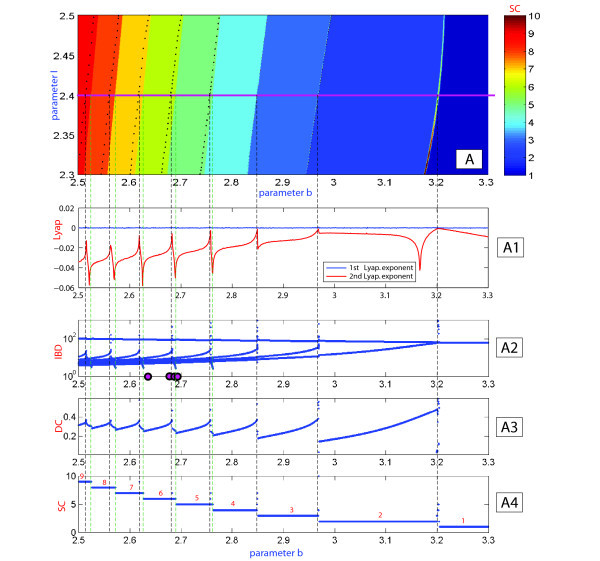
**(A)** Magnification of the (b,I)-SC diagram in Figure 3(A). **(A1)** First and second Lyapunov exponents plotted against the parameter *b*. Note that the second Lyapunov exponent of the bursting orbit raises to zero at the spike-adding transition and drops after. **(A2)** Interspike interval *vsb* at I=2.4. Last interspike intervals in a burst increase at the spike addition. This is an indication of the homoclinic bifurcation. **(A3)** Evolution of the duty-cycle and **(A4)** spike variability as *b* is varied.

**Fig. 6:**
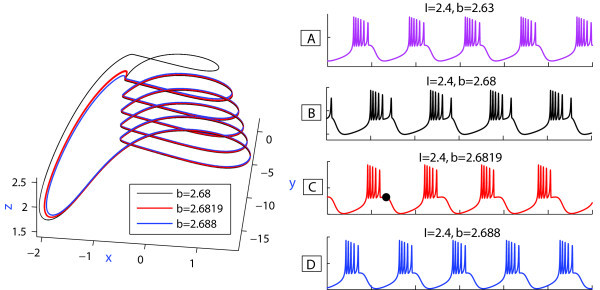
Left: three-dimensional [3D] projections of three bursting orbits passing by the saddle. An extra spike is gained by the orbit after it has switched the outgoing directions determined by the unstable separatrices of the saddle. **(A)-(D)** Waveforms of four bursting orbits, shown in the phase space of the model on the left, at spike-adding or deletion bifurcations for the indicated parameter values of *I* and *b*.

We have pointed out the changes in the color representation (value) of the duty-cycle in Figure 3(B). This variability can be due to two reasons: one is the change in the number of spikes per burst; the other reason is due to noticeable changes in time intervals between the spikes (given that the spike duration itself does not vary much). The answer is given by Figure 5 demonstrating the evolution of the temporal characteristics of bursting as the *b*-parameter is moved along the parametric cut I=2.4. More specifically, using Figure 5 we examine the dependence of the first and second Lyapunov exponents in Inset (A1), the evaluations of the interspike interval bifurcation-diagram in (A2), and the duty-cycle of bursting in (A3); finally, the spike-counting diagram in (A4) gives the number of spikes per burst as *b* is varied. The top of Figure 5 shows a magnification of the SC diagram in Figure 3(A), depicting the regular dynamics of the model, as the Lyapunov exponent stays non-positive on this pathway, corresponding to some periodic oscillatory activity in the model. The maximal Lyapunov exponent remains at zero, while the second Lyapunov exponent shows intervals of growth to zero alternating with those of rapid decrease. Observe that the peaks in the value of the second Lyapunov exponent occur at spike adding transitions, when the bursting orbit comes close to the saddle which is the threshold between the spikes and the quiescent segment of the former. Spike adding can be detected by the increase of the last interspike interval in the bifurcating bursting orbit that results automatically with an increase of the value of the the duty cycle orbit, and falls right after every spike addition transition. This tendency is clearly interpreted when one examines the changes in the voltage *x*-traces shown in Figure 6. Inset on the left depicts the evolution of the bursting orbit gaining an extra spike after it comes close by the saddle, and leaves along by the other unstable separatrix of the saddle. One notices from Figure 6 that the first two bursting orbits have six spikes in each burst, while the trace of the second (B) shows a prolonged interspike interval at the end of the burst (also revealed in Inset (A2) in Figure 5). The time interval prior the last spike grows to a point where the isolated spike disappears and is substituted by a short action potential (see the third orbit, where the black point indicates the local *x*-maxima of the short oscillation). This bursting orbit has five large spikes followed by a single short spike. After this short spike disappears the bursting orbit steadily exhibits five spikes. The process occurs at every spike-deletion bifurcation, or spike adding bifurcation, if instead the parameter *b* is decreased. 

 Indeed, the fact that the interspike interval grows by the end of the burst is a signature of square-wave bursters. Recall that these bursters are also code-named fold/homoclinic meaning that the spiking, slow-motion manifold is terminated through a homoclinic bifurcation of the saddle that occurs in the fast subsystem of the model, see Figure 2. The dwelling time of the phase point along the bursting orbit grows logarithmically fast the closer the point comes to the saddle [22]. The increase of the dwelling time is a generic phenomenon for all systems in a neighborhood of a saddle. What makes this phenomenon special for slow-fast systems is that the time scale of dynamics of the fast subsystem near the saddle turns out to be that of the slow subsystem, which gives rise to another peculiar phenomenon of ‘delayed loss of instability’ such that the phase point, previously turning around the spiking manifold, can be dragged along the middle, saddle branch $M_{\mathrm{eq}}$ of equilibria possibly all the way to the upper fold, provided that the timing is right, that is, the phase meets the saddle point right on the edge of the spiking manifold (lousily speaking, we encounter another kind of solutions broadly called ‘canards’, commonly characterized by the fact that a canard can follow an unstable branch of a slow-motion manifold). If the phase point reaches the edge before the saddle, it falls down to the hyperpolarized branch of $M_{\mathrm{eq}}$ to start a new cycle of bursting. If the phase point travels past the saddle, then it goes up along the other leading unstable separatrix of the saddle, makes another turn around $M_{\mathrm{lc}}$, resulting in the addition of an extra spike in the bursting orbit. Note that when the phase point does not approach the saddle, the model generates bursts with same number of spikes. Again, let us emphasize that such a spike adding mechanism is typical for square-wave or fold/homoclinic bursters; however, underlying mechanisms for spike adding can be completely different even in square wave bursters, and other neuronal models [19,24,40,42], including the leech heart interneuron model [17,18].

## 4 Screening the HR-model in the $\left(x_{0},I\right)$ and $\left(x_{0},\varepsilon \right)$-planes

**Fig. 7:**
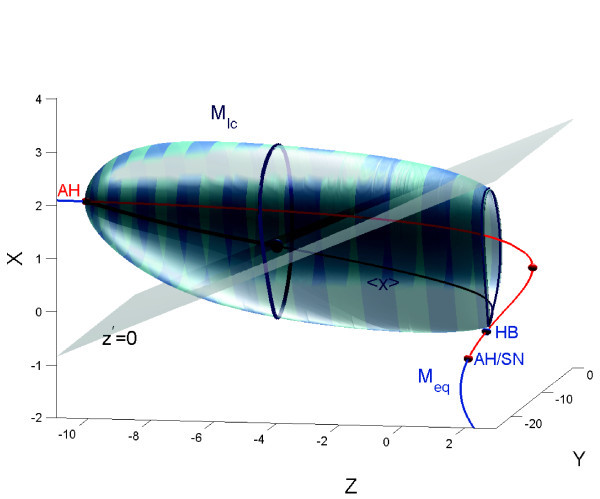
(3D version of Figure 2) Intersection point of the branch $M_{\mathrm{eq}}$ with the slow nullcline $\dot{z}=0$ yields the equilibrium state of the HR model at given $x_{0}$. The dark blue point is the center of the gravity of the stable periodic orbit of the HR model, which is depicted on the tonic spiking manifold $M_{\mathrm{lc}}$ at $x_{0}=1.8$. It is located around the intersection point of the slow nullcline $\dot{z}=0$ with the space curve 〈x〉 of the average *x*-values on each periodic orbit that foliate the spiking manifold $M_{\mathrm{lc}}$. The phase point, while turning around $M_{\mathrm{lc}}$, moves slowly toward the homoclinic edge ($\dot{z}>0$) as long as it stays above the slow nullcline, and goes backward ($\dot{z}<0$) after it lowers below the slow nullcline $\dot{z}=0$. When these opposite forces are canceled out over the revolution period, the phase point spins around the ‘center of the gravity’, that is, stays on the same periodic orbit. The variations of $x_{0}$ move the slow nullcline and thus make the periodic orbit slide along the manifold. When the slow nullcline $\dot{z}=0$ cuts through the unstable section of $M_{\mathrm{eq}}$ between HB, standing for homoclinic bifurcation in the fast subsystem and the fold AH/SN, the model becomes a burster.

 In this section we examine the dynamics of the model in response to variations of the slow parameter *ε*. In the neuroscience context, *ε* can be treated as the reciprocal of *τ*, which determines the (in)activation rate of the slow current in a neuronal model. For the sake of consistency we will first screen the model in the $\left(x_{0},I\right)$-parameter plane, while fixing b=3 and c=−3. Recall that the parameter $x_{0}$ moves the slow nullcline of the model up and down in the *x*-direction (see Figure 7). As the slow equation in (1) contains no *y*-variable, the plane in the (x,y,z)-phase space of the HR model, where the time derivative $\dot{z}$ vanishes, is the slow nullcline. One can see that $\dot{z}<0$ and $\dot{z}>0$ below and above this nullcline, respectively. Note that a simple round periodic orbit on the tonic spiking manifold, $M_{\mathrm{lc}}$, corresponds to regular tonic spiking activity in the model. The position of the periodic orbit on $M_{\mathrm{lc}}$ depends on where the slow nullcline $\dot{z}=0$ cuts through $M_{\mathrm{lc}}$. By changing $x_{0}$, we make the periodic orbit shift along the spiking manifold. More specifically it can be found around the intersection points of the slow nullcline with the average branch 〈x〉, see details in [2]. Tonic spiking remains regular until the periodic orbit stays away from the ‘homoclinic’ edge of $M_{\mathrm{lc}}$. 

As it was said previously the HR model describes one of the most typical configurations of slow manifolds for square-wave bursting oscillations. First of all, the configuration needs the distinct Z-shape for the quiescent manifold, $M_{\mathrm{eq}}$, with the lower branch corresponding to a hyperpolarized quiescent state of the neuron, while the upper unstable branch is surrounded by the spiking manifold, $M_{\mathrm{lc}}$, foliated by the stable limit cycles of the fast subsystem in the square bursting case. The branch regains stability in the case of plateau-like bursting. The manifold $M_{\mathrm{lc}}$ terminates through the homoclinic bifurcation that occurs in the fast subsystem in the square bursting case. Between the lower fold and this homoclinic point, the system has a hysteresis which gives rise to bursting. In the bursting regime, the phase point of the HR model switches repeatedly between the spiking, $M_{\mathrm{lc}}$, and quiescent, $M_{\mathrm{eq}}$, manifolds when it reaches their ends. In addition, both manifolds must be transient for the passing solutions of (1), that is, $M_{\mathrm{eq}}$ must be cut by the slow nullcline through the middle, saddle branch below $M_{\mathrm{lc}}$ and above the hyperpolarized fold point. This guarantees that $M_{\mathrm{lc}}$ is also transient for the trajectories of the model that coil around $M_{\mathrm{lc}}$ while translating slowly towards the edge, which corresponds to the aforementioned homoclinic bifurcation. Thus, the rapid jump from the lower point on $M_{\mathrm{eq}}$ towards $M_{\mathrm{lc}}$ indicates the beginning of the spiking period of a burst followed by the resting phase when the phase point drifts slowly along $M_{\mathrm{eq}}$ towards the fold, onto which it lands right after the homoclinic bifurcation. The number of complete revolutions of the phase point around the spiking manifold, $M_{\mathrm{lc}}$, gives the number of spikes within a burst, see Figures 2 and 6. Bursting in the model takes place as long as the slow nullcline hits $M_{\mathrm{eq}}$ between the points labeled HB, corresponding to the homoclinic bifurcation, and AH/SN standing for the singular Andronov-Hopf bifurcation in the full system, see Figure 7.

Thus, by varying $x_{0}$ we can make the model generate trains of bursts with various number of spikes. It is easy to see that the value of the small parameter *ε* determines the slow passage along both manifolds. So, halving *ε* should make bursts twice as long at least, with a doubled number of spikes. Note, too, that the duration of the quiescent phase should increase proportionally. As for variations of *I* are concerned, *I* moves, geometrically, the manifold horizontally in the 3D phase space of the model, in particular due to linearity of the slow equation in both variables. We show that because of that both *I* and $x_{0}$ act similarly on dynamics; of special interest here are transitions between the activity type of the model, which this section is focused on.

**Fig. 8:**
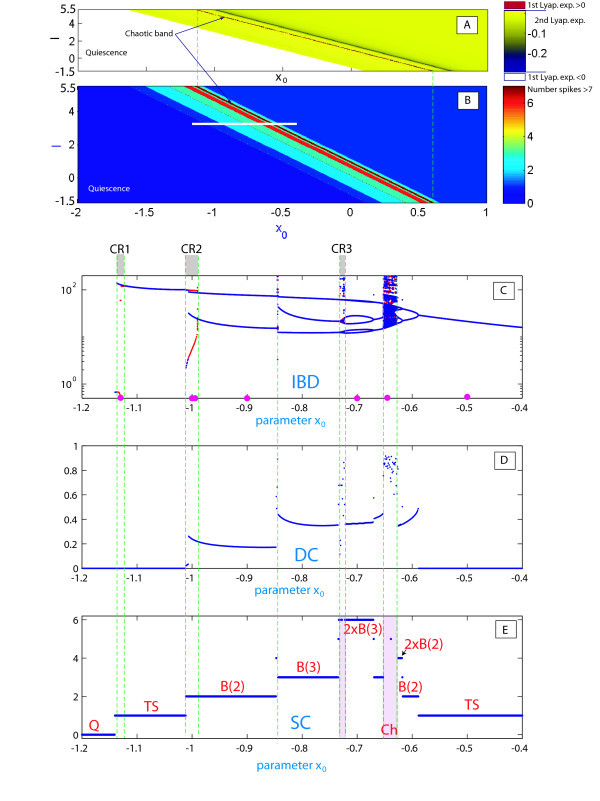
**(A)** 2D $\left(x_{0},I\right)$ Lyapunov exponents diagram at ε=0.01; **(B)** 2D $\left(x_{0},I\right)$ spike-counting diagram at ε=0.01; **(C)** 1D interspike-interval diagram *vs*$x_{0}$ for I=3.5; **(D)** Bursting duty-cycle dependence on $x_{0}$; **(E)** Spike variability per burst is plotted against $x_{0}$.

 Figure 8 demonstrates the 2D $\left(x_{0},I\right)$-spike counting diagram with ε=0.01 and the same picture using the first and second Lyapunov exponents. The diagram reveals a diagonal plot structure foliated by homogeneous bands. This suggests that variations of parameters *I* and $x_{0}$ cause similar responses in the model. On the band structure there is a thin band of chaotic motion located inside the band of high number of spikes, as remarked by the positive value of the maximum Lyapunov exponent. To gain insight into the band structure, we examine the evolutions of dynamics of the model as only $x_{0}$ is varied at fixed current input I=3.5. At smaller values of the parameter, the model exhibits tonic spiking first, see Insets (C) and (E) of Figure 8 presenting the interspike bifurcation diagram (IBC), and the spike counting (SC) diagram. As the parameter is decreased further toward the bursting zone, the model enters a period-doubling cascade leading to chaos [20,43]. In Figure 8(E) we see groups of spikes but without a clear bursting structure (bursting orbits with *n* spikes that doubles the period are denoted by 2×B(n)). Next, the model has a chaotic orbit (in region Ch), evolving into a compact chaotic region, which undergoes a boundary crisis, widening drastically the size of the chaotic attractor (also reported in [44,45]). As $x_{0}$ is decreased further, chaos terminates through another boundary crisis due to intermittency originating from a fold bifurcation. The model now generates regular trains of bursts. The corresponding bursting orbit further undergoes a series of period-doubling and period-halving bifurcations, before it steps into a cascade of spike deletion bifurcations, eventually leading to quiescence (Q) on the left hand side of of the $x_{0}$-parametric pathway, after the Andronov-Hopf bifurcation. 

**Fig. 9:**
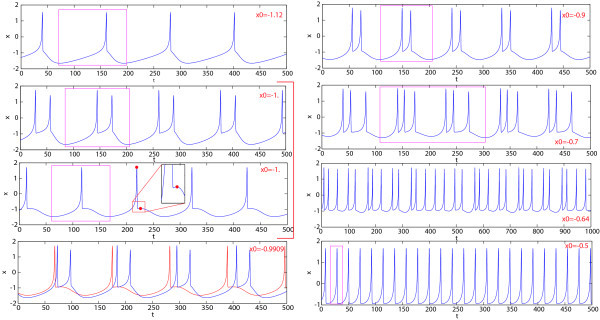
Tonic spiking and bursting *x*-traces at selected points on the *b*-parameter passway (shown in magenta) across the diagram in Figure 8(C) showing several stages of spike addition transitions.

**Fig. 10:**
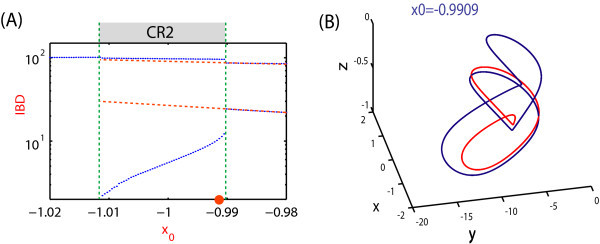
**(A)** Magnification of the interspike bifurcation-diagram (IBD) for I=3.5 in the bistable region CR2 from Figure 8. It reveals two coexisting bursting orbits (shown in red and blue) at $x_{0}=-0.9909$ in the phase space of the model: the split between the solutions takes place near the saddle after the orbits departs in the direction determined by the unstable separatrices. **(B)** Two coexisting orbits corresponding to single and duplet spiking.

 Figure 9 demonstrates the waveforms of the bursting solutions at selected points (shown in magenta) in Inset (C) of Figure 8. One complete period of each waveform is squared out within a red box. Thus, we have the following orbits: a spiking orbit for $x_{0}=-1.12$, two bistability points for $x_{0}=-1$ and −0.9909, B(2)-orbit (bursting with two spikes) for $x_{0}=-0.9$2×B(3)-orbit for $x_{0}=-0.7$, chaotic orbit for $x_{0}=-0.64$ and again a regular spiking orbit at $x_{0}=-0.5$ of a smaller period compared to that at $x_{0}=-1.12$. A quite interesting observation can be deduced from Figure 8(C). Namely, there are several regions of bistability where there are coexisting stable periodic orbits. These regions, labeled as CR1, CR2 and CR3, are marked by using two colors in the IBD-diagram in Inset (C). The middle bistability region, CR2, is expanded in Figure 10(A), and it reveals that there co-exist two distinct bursting orbits at $x_{0}=0.9909$. The 3D phase projections and the corresponding waveforms, with single spikes and spike duplets (B(2)), are shown in Figures 10(B) and 9, respectively. Figure 9 presents two plots of the waveforms for the co-existing orbits at $x_{0}=-1$; the black point in Figure 6(C), indicates the local *x*-maxima of the short waveform. As was pointed out earlier such a coexistence of (n)-spike and (n+1)-spike bursts is a typical phenomenon for square-wave bursters at the spike adding or deletion transitions due to ‘the delay loss of instability’ that occurs along the saddle, threshold branch of $M_{\mathrm{eq}}$ that separates depolarized and hyper-polarized states of the neuron model [41]. Recall that theoretically, due to the equal time scale dynamics of the fast subsystem near the saddle and the slow equation because of small *ε*, the phase point can be dragged along the saddle branch up to the upper fold on $M_{\mathrm{eq}}$. Interestingly enough, the range of bistability zones will shrink as the value of *ε* is increased. We would like to point out that multistability is a by-product of the nonlinearity in the system. Multistability has been reported in several neuronal systems both experimentally and computationally, including individual neurons and their models, as well as in neural networks and multifunctional central pattern generators [1,46]. Multistability is of great interest for neuroscience as it can potentially enhance the flexibility of the nervous systems, decision making processes, and explain various nervous pathologies caused by sudden changes in system’s states. 

**Fig. 11:**
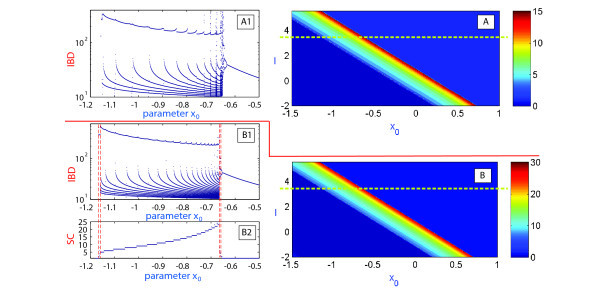
**(A)** 2D spike-counting diagram projected to the plane $\left(x_{0},I\right)$ at ε=0.002; **(A1)** interspike-duration bifurcation diagram for I=3.5. **(B)** 2D spike-counting plot in the plane $\left(x_{0},I\right)$ for ε=0.001; **(B1)** interspike-duration bifurcation diagram for I=3.5 and **(B2)** 1D spike-counting diagram for I=3.5.

**Fig. 12:**
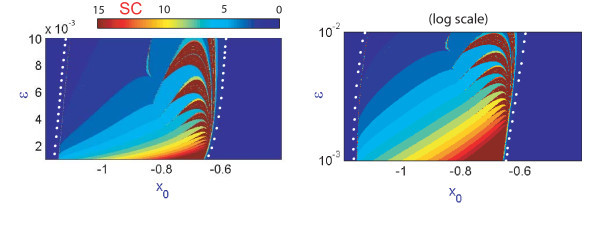
$\left(x_{0},\varepsilon \right)$ spike-counting diagram in the linear and logarithmic scales in *ε*. Shown in blue and red are regions of passive silence and intense spiking activities.

Another peculiar observation related to the band structure in the $\left(x_{0},I\right)$-parametric plane is concerned with the regions of high sensitivity to small variations of the control parameter $x_{0}$, whereas the overall band structure seems to be quite robust, or self-similar in *I*. This brings in one remaining question we would like to addresses in the paper: will this property of self-similarity of the band structure persist for smaller values of *ε*? Our findings are summarized in Figure 11 which demonstrates the bifurcation diagrams for two values: ε=0.002 and ε=0.001. They confirm that the band structure does persist and show predictably regions with larger number of spikes per bursts, especially for ε=0.001 (compare the SC plot in 1D and 2D diagrams). To study the genesis of the band structure we also built the corresponding $\left(x_{0},\varepsilon \right)$-diagrams shown in Figure 12. Although both diagrams represent the same data of the spike-counting, the diagram on the right in Figure 12 is given in a logarithmic scale to demonstrate that the self-similarity property of the band structure is exponential in *ε*. We can deduce from the diagrams that the most interesting, in terms of dynamics, region is contained between two curves (indicated by white dots). Within this region there are several diagonal bands corresponding to bursting orbits with different numbers of spikes. This plot explains the fact that a small *ε*-parametric cut will nevertheless reveal the band structure, observed in Figures 8 and 11, thus confirming that either bifurcation parameter *I* or $x_{0}$ can be equality singled out to perform the bifurcation analysis of the Hindmarsh-Rose model. 

## 5 Conclusions

 As of today, the Hindmarsh-Rose remains justifiably one of the most popular mathematical models, that describes, qualitatively well, the dynamics of a certain class of neuronal models derived using the Hodgkin-Huxley formalism. The model has been carefully analyzed using various mathematical and computational tools, and has been viewed though the prisms of the advanced bifurcation theory, geometric methods of slow-fast dynamical systems to reveal multiple peculiar qualitative features. Various brute force approaches [27,28] have been applied to the model to reveal its quantitative or metric properties though the examination of the Lyapunov exponent spectrum and the number of spikes per period.

 In this paper we have tested other computationally effective tools tailored specifically for models originating in neuroscience, including 1D and 2D parametric screenings of the complex dynamics of the model aimed to bridge notions common for neuroscience with the accurate mathematics-based findings. Our computational toolkit includes several methods for examining of temporal characteristics of a single variable, *x*, treated as a voltage across the cell membrane, or more specifically, the corresponding voltage waveforms. The list includes the spike-counting approach, the evaluations of inter-spike interval, and duty cycles of bursting, which is a ratio of the burst duration (active phase) over the total burst period. We confirmed our findings based on these methods with ‘calculus’-based simulations for the whole spectrum of the Lyapunov exponents calculated for solutions of a system of ordinary differential equations like the Hindmarsh-Rose model. Our verdict is that both approaches consistently demonstrate very good agreements. The methods allow us to give detailed explanations for various global bifurcations in the model, including various spike-addition/deletion cascades, phenomena of bistability, and various transitions between types of bursting: square-wave and plateau-like. This ensures that this toolkit of combined neuroscience-native methods and calculi-based algorithms will yield effective and timely insights for pilot studies of new, previously unidentified, in sense of dynamics and bifurcations, models of individual neurons, as well as other cells, such as myocytes - cardiac tissue cell. We showed in [26] that the proposed methods could provide the bifurcation details and add even problem-specific nuances into regulation control of temporal characteristics of realistic interneuron models of the leech. The evident advantage of the approach is its nativeness for the neuroscience community. Last but not least, it should emphasized that a 2D parameter sweeping of the model shown in Figures 3 and 4 takes about 10-20 times faster than the sweeping based on the Lyapunov exponents spectrum (Figure 5). A drawback of the approach is that it needs to be corrected in cases where the model is multistable; this remains common weakness of all straightforward methods unless one uses randomized initial conditions and longer transients that could overall substantially prolong the simulation time.

 It is in our future plans to broaden the applicability of these proposed computational tools for studies of neuronal networks, especially for multifunctional central pattern generators comprised of several neurons [2,46]. Such a multifunctional CPG is capable of generating multiple bursting rhythms at quite different time scale. It was shown recently in [3] that bursting outcomes of a multistable 3-cell network are determined by the duty cycle of bursting. Moreover, the longest bursting cell plays the role of the pacemaker of the network [47]. Note also that bursting network can be composed of individually tonic spiking cells that while inhibiting, even weekly, each other can create various bursting outcomes of the network as a whole. This indicates directly that such a cell, whether tonic spiking or bursting, is to be close to the boundary separating the activity types in the parameter space of distinct interneurons [25,26], in particular to control effectively the temporal characteristic of bursting, regular or chaotic. In the light of saying, it is evident that the tools native to neuroscience paradigms are suited more appropriately for studying bursting metamorphoses in networks and the side-by-side comparison of the results of mathematical and experimental studies using the common jargon. This computational toolkit shall bring us closer to the targeted goal - to build realistic and adequately responding models of concrete functional CPGs with specific time scales, phase locked states between synergetically coupled neurons with plausible bursting characteristics.

## Competing interests

The authors declare that they have no competing interests.
